# LONG-TERM IMPACTS OF A LIFESTYLE INTERVENTION IN OLDER ADULTS WITH DIABETES AND OVERWEIGHT OR OBESITY: LOOK AHEAD

**DOI:** 10.1093/geroni/igae098.0385

**Published:** 2024-12-31

**Authors:** Peter Huckfeldt, Charles Semelka, John Jakicic

**Affiliations:** Chair; Co-Chair; Discussant

## Abstract

Mid-life weight loss and maintenance is often recommended as a means to improve health later in life, however its long-term benefits have not been rigorously established. The Action for Health in Diabetes (Look AHEAD) randomized controlled trial compared an intensive lifestyle intervention (ILI) designed to induce and maintain a 7% weight loss through caloric restriction and increased physical activity with a control condition of diabetes support and education (DSE) in 5,145 individuals aged 45-76 years with type 2 diabetes and overweight/obesity over up to 11 years of follow-up beginning in 2001. Although the ILI group had greater initial improvements in cardiovascular disease risk factors compared to DSE, there were no overall differences in the incidence of the trial’s primary and secondary cardiovascular outcomes between the ILI and DSE groups. However, ILI did have a positive impact on diabetes control and complications, depression, physical health–related quality of life, and health care use and costs. Subsequently, the Look AHEAD trial transitioned to an in-person observational study in 2012 to assess the long-term benefits and potential harms of an ILI, which continued for 10 years before transitioning to phone-only observational period in 2022 – the Look AHEAD Aging study. This symposium will describe the long-term impact of the lifestyle intervention and the effect of diabetes-related risk factors on self-reported vision and hearing (Espeland and colleagues), nutritional risk (Houston and colleagues), self-reported disability in basic activities of daily living, instrumental activities of daily living, and mobility (Semelka and colleagues), and multi-morbidity (Davis and colleagues).

